# Exosome-based biomimetic nanoparticles targeted to inflamed joints for enhanced treatment of rheumatoid arthritis

**DOI:** 10.1186/s12951-020-00675-6

**Published:** 2020-08-20

**Authors:** Feili Yan, Zhirong Zhong, Yao Wang, Yue Feng, Zhiqiang Mei, Hui Li, Xiang Chen, Liang Cai, Chunhong Li

**Affiliations:** 1 Department of Pharmaceutical Sciences, School of Pharmacy, Southwest Medical University, 3-319 Zhongshan Road, 646000 Luzhou, Sichuan People’s Republic of China; 2 Department of Nuclear Medicine, The Affiliated Hospital of Southwest Medical University, 3-319 Zhongshan Road, 646000 Luzhou, Sichuan People’s Republic of China; 3 Nuclear Medicine and Molecular Imaging Key Laboratory of Sichuan Province, 646000 Luzhou, Sichuan China; 4 The Research Center for Preclinical Medicine, Southwest Medical University, 646000 Luzhou, Sichuan China; 5 Engineering Research Center in Biomaterials, Sichuan University, 610064 Chengdu, Sichuan People’s Republic of China

**Keywords:** Dexamethasone sodium phosphate, Biomimetic, Exosomes, Folic acid, Rheumatoid arthritis

## Abstract

**Background:**

Glucocorticoids (GCs) show powerful treatment effect on rheumatoid arthritis (RA). However, the clinical application is limited by their nonspecific distribution after systemic administration, serious adverse reactions during long-term administration. To achieve better treatment, reduce side effect, we here established a biomimetic exosome (Exo) encapsulating dexamethasone sodium phosphate (Dex) nanoparticle (Exo/Dex), whose surface was modified with folic acid (FA)-polyethylene glycol (PEG)-cholesterol (Chol) compound to attain FPC-Exo/Dex active targeting drug delivery system.

**Results:**

The size of FPC-Exo/Dex was 128.43 ± 16.27 nm, with a polydispersity index (PDI) of 0.36 ± 0.05, and the Zeta potential was − 22.73 ± 0.91 mV. The encapsulation efficiency (EE) of the preparation was 10.26 ± 0.73%, with drug loading efficiency (DLE) of 18.81 ± 2.05%. In vitro study showed this system displayed enhanced endocytosis and excellent anti-inflammation effect against RAW264.7 cells by suppressing pro-inflammatory cytokines and increasing anti-inflammatory cytokine. Further biodistribution study showed the fluorescence intensity of FPC-Exo/Dex was stronger than other Dex formulations in joints, suggesting its enhanced accumulation to inflammation sites. In vivo biodistribution experiment displayed FPC-Exo/Dex could preserve the bone and cartilage of CIA mice better and significantly reduce inflamed joints. Next in vivo safety evaluation demonstrated this biomimetic drug delivery system had no obvious hepatotoxicity and exhibited desirable biocompatibility.

**Conclusion:**

The present study provides a promising strategy for using exosome as nanocarrier to enhance the therapeutic effect of GCs against RA.
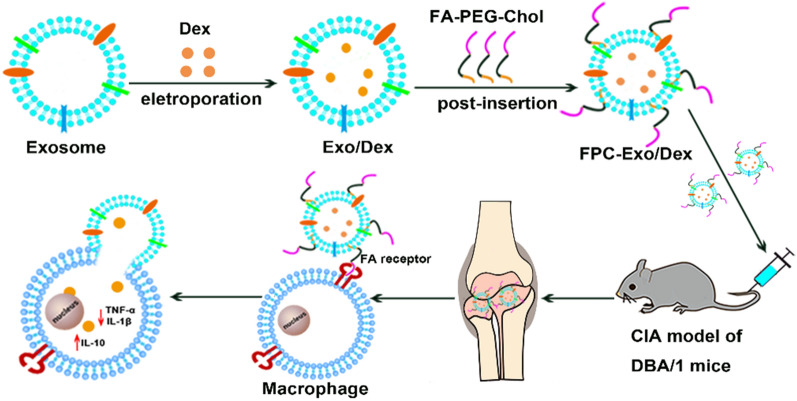

## Introduction

Rheumatoid arthritis (RA) is a chronic autoimmune disease whose pathophysiology is unclear [1]. As it progresses, articular cartilage and bone are destroyed, which can cause disability eventually [2, 3]. This progression may be driven by pro-inflammatory cytokines such as tumor necrosis factor (TNF)-α and the interleukins (IL)-1β and IL-6, as well as the inflammatory mediators inducible nitric oxide synthase and epoxidase [4, 5]. Glucocorticoids (GCs), the most widely used first-line drugs against RA, can control inflammation and relieve pain quickly by inhibiting the secretion of pro-inflammatory cytokines and up-regulating anti-inflammatory protein IL-10 [6, 7], which further inhibits the secretion of pro-inflammatory cytokines. However, long-term use of systemic, high-dose glucocorticoids is associated with serious adverse effects, such as decreased immunity, hyperglycemia and osteoporosis [8].

Delivering anti-RA drugs within nanoparticles may help target the drugs to inflamed tissues, thereby improving therapeutic efficacy and reducing adverse effects [8, 9]. Nanoparticles can accumulate at inflammatory sites through a process known as “extravasation through leaky vasculature and subsequent inflammatory cell-mediated sequestration” (ELVIS), which is analogous to the “enhanced permeability and retention” (EPR) effect that can lead nanoparticles to accumulate in solid tumors.

Indeed, targeted delivery of glucocorticoids against RA has already been achieved using liposomes, physically encapsulated micelles and polymer nanoparticles [2, 10–12]. However, liposomes are poor at evading host immune system clearance, and they show instability and short circulation time [13, 14]. Physically encapsulated micelles are unstable in circulation because they are diluted and they can interact with proteins and other components of plasma [2, 3]. Polymer nanoparticles may be more stable than liposomes and physically encapsulated micelles, but their long-term biocompatibility and safety pose challenges [2, 5]. Consequently, developing more reliable and powerful nanocarriers is highly desired.

Exosomes have emerged as an alternative to those exogenous nanoparticles [15, 16]. These membrane-enclosed vesicles with sizes of 40–150 nm are naturally secreted by various cell types, which endow them with fascinating natural properties such as low cytotoxicity, non-immunogenicity, desirable biocompatibility, specific targeting capacity and prolonged systemic circulating ability [15, 17–19]. These superior properties making them ideal drug delivery nanocarriers. Moreover, exosome-based nanoparticles drug delivery has been successfully applied in loading various drugs for different diseases treatment, such as cancer, Parkinson, renal and brain inflammation, but has been rarely used in RA so far [19–22]. However, the exosome-based drug delivery existed flaw in selective accumulation to target sites in vivo [21–23].

Study on the pathological microenvironment of RA suggested that inflamed areas contain abundant activated macrophages that express folic acid receptors (FRs) on their surface, particularly FRβ [24, 25]. Therefore, we reasoned that it may be effective to construct exosome-based nanoparticles coating with folic acid (FA) for enhancing accumulating ability by active targeting effect to FRβ in vivo. Therefore, we used exosomes to encapsulate dexamethasone sodium phosphate (Dex), one of the most frequently used GCs to treat RA in clinical, to obtain Exo/Dex nanoparticle firstly. Then, we modified it with FA-polyethylene glycol (PEG)-cholesterol (Chol) compound (FPC) to prepare FPC-Exo/Dex active targeting drug delivery. Meanwhile liposome drug delivery system (Lip/Dex) was fabricated for comparative study to Exo/Dex in this research. Properties of these nanoparticles were characterized in vitro. Then the internalization and anti-inflammatory effects to lipopolysaccharide-activated RAW264.7 cells were examined. Furthermore, their biodistribution, therapeutic efficacy against RA and in vivo safety were studied in mice with collagen-induced arthritis (CIA).

## Material and methods

### Materials

Dexamethasone sodium phosphate was supplied by Solarbio Science & Technology (Beijing, China). Methanol and acetonitrile (HPLC grade) were purchased from Kelong Chemical Reagent Factory (Chengdu, China). Rabbit monoclonal antibodies against CD63 or CD9 as well as horseradish peroxidase-conjugated goat anti-rabbit IgG were purchased from Abcam (UK). SDS-PAGE kits and polyvinylidene fluoride membranes were obtained from Sigma (USA). Complete and incomplete Freund’s adjuvant and bovine type II collagen were acquired from Chondrex (USA). ELISA kits were from Thermo Fisher (Austria). Fluorescent dyes PKH26 and PKH67 kits were purchased from Beijing Baiao Laibo Technology; 1, 1′-Dioctadecyl-3, 3, 3′, 3′-tetramethylindodicarbocyanine perchlorate (DID), 3-(4,5-dimethylthiazol-2-yl)-2,5-diphenyltetrazolium bromide (MTT), FITC-labeled phalloidin DAPI and Lipopolysaccharide (LPS) were supplied by Beijing Solarbio Science & Technology. Other reagents were analytical grade.

### Cells and animals

RAW 264.7 murine macrophages and human umbilical vein endothelial cells (HUVEC) (Chinese Academy of Sciences, Shanghai, China) were cultured in DMEM/HIGH GLUCOSE medium (Hyclone, USA) with 10% fetal bovine serum(FBS) (Gibco, USA) and 1% (v/v) penicillin/streptomycin (Hyclone, USA).

Male DBA/1 mice (8 weeks old) were purchased from Charles River (Beijing, China). All animal experiments were in accordance with The Animal Ethics Committee of Southwest Medical University.

### Isolation and characterization of exosomes

When RAW 264.7 cultures reached the logarithmic phase of growth, cells were switched to FBS-free medium for 24 h, after which the medium was collected and replaced with fresh FBS-free medium. The cultures were incubated for another 24 h, then the medium was collected again. The two volumes of collected medium were pooled, exosomes were isolated using a gradient centrifugation protocol [20] with some modification. Firstly, the medium was centrifuged at 2000 g for 10 min and then at 10,000*g* for 30 min to remove cellular debris. Next, the supernatant was concentrated to about 30% of the original volume at 2000*g* for 8 min using ultrafiltration tube (MWCO = 10,000). Finally, the supernatant was centrifuged at 120,000* g* for 70 min in an ultracentrifuge (QPTimaMAX-XP Ultra-High, Beckman Coulter, USA). The pellets were washed with large volume cold phosphate-buffered saline (PBS) and centrifuged at 120,000* g* for 70 min again to ensure maximal exosome purity. All centrifugation procedures were performed at 4 °C. The pellet was re-suspended in PBS and stored at − 80 °C. The amount of exosomes was estimated using a Bradford assay (Bio-Rad Laboratories, Shanghai, China).

Size, polydispersity index (PDI) and zeta potential of purified exosomes were determined using dynamic light scattering (Malvern Zetasizer Nano ZS90, Malvern Instruments, UK). Their morphology was examined using transmission electron microscopy (HT7700, Hitachi, Japan). The presence of CD63 and CD9 on the exosome surface were measured by western blotting. These proteins serve as markers of exosomes derived from mammalian cells [18, 26].

### Preparation of Exo/Dex and FPC-Exo/Dex

To load Dex into exosomes, exosomes (100 μg) were mixed with Dex (300 μg) in PBS with the concentration of trehalose was 80 nM, which was added to avoid the aggregation of exosomes during electroporation. The mixture was subjected to electroporation at room temperature using a double poring pulse (200 V, 5 ms) and transfer pulse of five pulses (20 V, 50 ms) in a 1-cm electroporation cuvette and a NEPA21 Type II electroporater (NEPA genes, Tokyo, Japan). Then un-encapsulated Dex was removed by ultracentrifugation (100,000 g*,* 60 min, 4 °C). The Dex-loaded exosomes (Exo/Dex) were resuspended in PBS and incubated in 37 °C for 1 h to restore the membrane.

The FA-PEG-Chol conjugate was prepared as described in Supplementary Materials. The Chol end of FA-PEG-Chol was inserted into the lipid bilayer membrane of Exo/Dex by post-insertion [22]. Exo/Dex and FA-PEG-Chol ligands were mixed in a mass ratio of 1:5 and incubated at 37 °C for 2 h. Free ligands were removed by centrifugation at 3000 g for 10 min, giving rise to FPC-Exo/Dex. The amount of FA incorporated was determined by comparison of the UV_285_ value to a standard curve of folic [27, 28].

### Preparation of Dex-loaded anion liposomes (Lip/Dex) as control group

Dex-loaded anionic liposomes were prepared in order to compare with exosome-based drug delivery systems. Anionic liposomes were prepared using ethanol injection as described [29]. Firstly, 40 mg of yolk lecithin, 10 mg of cholesterol and 10 mg of Dex were dissolved in 3 mL ethanol, and the solution was slowly injected into 5 mL PBS (pH 7.4) while stirring. The solution was slowly stirred under bath conditions at 40 °C for 2 h, and the solution was filtered through 0.45 μm and 0.22 μm membrane successively, yielding Lip/Dex.

### Characterization of different Dex preparations

Size, PDI and Zeta potential of all preparations were determined using dynamic light scattering, and morphology of FPC-Exo/Dex were examined by transmission electron microscopy. Encapsulation efficiency (EE) and drug loading efficiency (DLE) were measured using high-performance liquid chromatography (HPLC). Briefly, each formulation was divided into two equal portions, one of which was demulsified with 10% methanol, and the amount of total drug (W_t_) was measured by HPLC. The free drug in the second portion (W_e_) was pelleted by ultracentrifugation and weighed. EE was calculated using the equation EE = W_e_/W_t_ × 100%. EE of Lip/Dex was determined in the same way.

The amount of exosomes (W_s_) was estimated by BCA assay to calculate DLE according to the equation DLE = W_e_/ (W_t_ + W_s_) × 100%. An equal amount of Lip/Dex was dried and weighed (W_p_) to calculate DLE according to the equation DLE = W_e_/W_p_ × 100%.

### In vitro cumulative drug release study

FPC-Exo/Dex, Exo/Dex, Lip/Dex, or free Dex (40 μg of Dex contained in all preparations) were added to 1 mL PBS (pH 7.4 or pH 6.0) in a dialysis bag with a molecular weight cut-off of 3000 Da. The bag was placed in 30 mL PBS and shaken at 37 °C at 1000 rpm. At predetermined time points, 200 μL of release medium was collected and immediately replaced with an equal volume of fresh medium. Dex concentration was determined by HPLC, and the cumulative amount released was calculated.

### Toxicity assay of nanoparticles by MTT

RAW264.7 cells and HUVEC in logarithmic growth phase were digested into single-cell suspensions and seeded in 96-well plates at 1 × 10^4^ cells per well, then incubated at 37 °C overnight. FPC-Exo/Dex, Exo/Dex and Dex were prepared in culture medium without serum or antibiotics, and 200 μL of each preparation was added to wells at Dex concentrations of 5–25 μg/mL. After 24 h, the medium was discarded, and 20 μL of MTT (5 mg/mL) solution and 180 μL of complete medium were added. Cells were cultured for another 4 h, then medium was replaced with 150 μL DMSO and cultures were shaken for 15 min at 37 °C. Absorbance at 490 nm was measured using a Varioskan Flash microplate reader (Thermo Fisher, USA). Relative cell viability was calculated using the equation: Cell viability = (sample − blank) / (negative control − blank) × 100%.

### Cellular uptake study by flow cytometry and confocal laser scanning microscopy

Lip/Dex, Exo/Dex and FPC-Exo/Dex were labeled with PKH67 or PKH26 according to the dye manufacturer’s protocol. PKH67-labeled Dex formulations were incubated for 2 h with resting or lipopolysaccharide (LPS)-activated (stimulated for 24 h with LPS at a final concentration of 100 ng/mL) RAW264.7 cells. Uptake by cells was measured using a Verse cytometer (BD, USA).

In order to visually observe the situation of the formulations entering the cell, the three Dex formulations labeled by PKH26 were incubated with resting or LPS-activated RAW264.7 cells for 2 h, then we analyzed endocytosis using confocal laser scanning microscopy (Leica SP8, Germany) after staining the cytoskeleton with FITC-phalloidin and the nucleus with DAPI [18].

### Anti-inflammatory effects of nanoparticles to LPS-activated RAW264.7 cells

RAW264.7 cells were seeded into a 24-well plate at a density of 5 × 10^5^ cells per well and allowed to adhere, then stimulated for 24 h with LPS at a final concentration of 100 ng/mL. The medium was replaced with fresh medium containing FPC-Exo/Dex, Exo/Dex, Lip/Dex or free Dex at a final Dex concentration of 20 μg/mL. Negative control wells were incubated in culture contain PBS. After 24 h, the culture medium was collected, centrifuged at 2000 g for 5 min, and the supernatant was assayed for TNF-α, IL-β and IL-10 using ELISA kits according to the manufacturer’s instructions [21].

### Establishing Mouse model of collagen-induced arthritis (CIA)

Bovine type II collagen was thoroughly emulsified with an equal volume of complete Freund’s adjuvant by vortex, and 100 μL emulsion was administered intradermally at the base of the mouse tail. After 21 days, mice received an intradermal booster injection of type II collagen with an equal volume of incomplete Freund’s adjuvant.

### Biodistribution of nanoparticles in CIA mice

A total of 12 CIA mice were randomly divided into 4 groups (3 animals per group), which were intravenously administered DID-labeled Lip/Dex, Exo/Dex, FPC-Exo/Dex, or free DID (1 μg DID per mouse). At 1, 4, 8, and 24 h later, mice were anesthetized with 10% chloral hydrate (0.04 mL per 10 g) and analyzed using the IVIS® Spectrum system (Caliper, Hopkinton, MA, USA).

At 24 h, mice were euthanized and the blood, heart, liver, spleen, lung, and kidney were removed. Blood was sampled and centrifuged at 3000 *g* for 7 min to obtain plasma. Fluorescence of plasma and organs was measured using the IVIS® Spectrum system (PerkinElmer, USA).

### Measurement of weight, paw thickness, foot volume and articular index (AI) score of CIA mice

On day 21 after the booster immunization, CIA mice were randomly assigned to five groups (3 animals per group) and injected intravenously with free Dex, Lip/Dex, Exo/Dex or FPC-Exo/Dex at a Dex dose of 1.2 mg/kg in all cases. Negative control mice were injected with the same volume of Saline. The first injection was delivered on day 21, and then once every four days for a total of four injections. AI scores were determined for each limb as described [8]. Body weight, hind paw thickness and foot volume were measured every 3 days during treatment. Foot volume is measured with drainage method.

### Micro-computed tomography (Micro-CT) analyses of articular bone

After mice were sacrificed, the left hind limbs were removed and immediately fixed in 4% paraformaldehyde for 48 h. Then the microstructure of each limb was analyzed using a SIEMENS Inveon PET/CT computed tomography system (SIEMENS, Germany) with the following parameters: voltage, 80 kV; current, 500 μA; exposure time, 1800 ms; total rotation, 220°; and projections, 120 sheets. A region of interest (ROI) of the trabecular bone within the calcaneus was defined by aligning the calcaneus bone along the sagittal plane using the Data Viewer, starting 0.2 mm away from the epiphyseal plate and continuing for 40–50 slides (1 mm) [30, 31]. The bone mineral density (BMD), percent bone volume (BV/TV), bone surface density (BS/BV), trabecular thickness (Tb.Th), trabecular number (Tb.N) and trabecular spacing (Tb.Sp) of the ROI were calculated using SIEMENS Inveon Research Workplace software 4.2.

### Histological evaluation of joint tissues

Ankle joints were dissected from each group and fixed in 4% paraformaldehyde for 48 h, and then decalcified in 10% neutral EDTA solution for 15 days at room temperature. Then decalcified tissue was embedded in paraffin. Thin Sects. (5 μm) were cut and stained with hematoxylin–eosin (H&E) or safranin O (SO) combines with chondroitin sulfate to stain articular cartilage red [32]. An H&E score from 0 to 3 was determined for each of the following aspects: inflammatory cell infiltration, synovial tissue proliferation, fibrous tissue hyperplasia, and macrophage infiltration. Then all these scores were summed for a given ankle joint, and the overall scores for all ankle joints were summed to obtain H&E scores for a given animal.

### Evaluation of inflammatory cytokines in serum

Blood samples were collected from mice on day 45 after induction of CIA. Serum levels of inflammatory cytokines TNF-α, IL-1β and IL-10 were measured using ELISA kits according to the manufacturer’s instructions.

### In vivo safety evaluation of nanoparticles in CIA mice

Aspartate transaminase (AST) and alanine transaminase (ALT) levels of serum collecting from different treatment groups were assayed using a commercial kit (Nanjing Institute of Biological Engineering, Nanjing, China) according to the manufacturer’s protocol.

### Statistical analysis

Statistical analysis was performed using GraphPad Prism 6.0 (GraphPad Software, La Jolla, CA, USA). Statistical comparisons were performed by one-way ANOVA (Dunnett's multiple comparisons test) for multiple groups, except for the analyses in Figs. 4b and  6b, which were performed using two-way ANOVA. Differences associated with *p* < 0.05 were considered significant. All results were expressed as mean ± SD.

## Results

### Characterization of exosomes

**Fig. 1:**
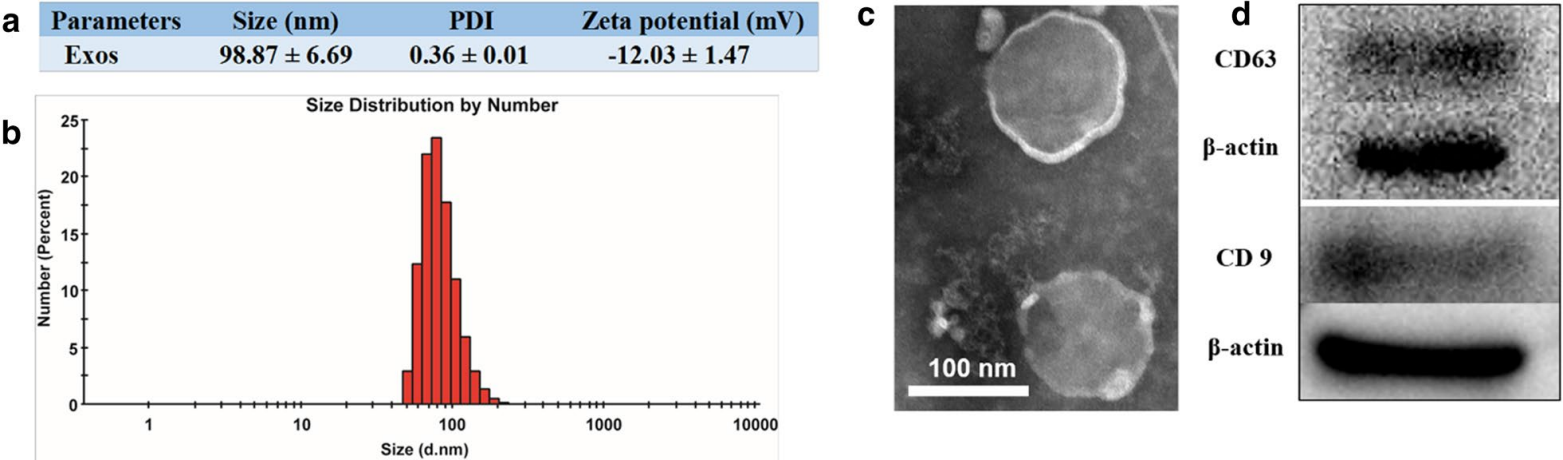
Characterization of exosomes. **a** Size, PDI and zeta potential of Exos. **b** Size distribution of Exos. **c** Transmission electron micrographs of Exos. **e** Western blot to confirm the expression of CD9 and CD63

Exosomes (Exos) showed an average size of 98.87 ± 6.69 nm, PDI of 0.36 ± 0.01, and zeta potential of − 12.03 ± 1.47 mV (Fig. 1a, b). Consistent with these results, transmission electron microscopy displayed that exosomes were round-shaped nanovesicles surrounded by membrane, with a diameter of approximately 100 nm (Fig. 1c). Ultimately, 2.20 ± 0.44 μg of exosomes (based on protein content) could be purified from 1 mL of culture medium. Western blot experiments showed that the exosomes contained the marker proteins CD9 and CD63 (Fig. 1d).

### Structure of FA-NHS and FA-PEG-Chol

^1^H-NMR showed signals at 6.93 ppm and 7.60 ppm belonging to the *para*-aminobenzoic acid moiety in FA, as well as signals at 4.21–4.30, 1.79–2.05 and 2.20–2.31 ppm belonging to the α-, β-, and γ-CH_2_-protons of the glutamic acid moiety of FA (Additional file 1: Fig. S1). The signal at 2.44–2.49 ppm was attributed to the proton of the NHS group.

^1^H-NMR of FA-PEG-Chol confirmed the formation of the conjugate, showing principal peaks related to FA (8.53–8.78, 7.73–7.65, 5.60–5.52, 4.64–4.53 ppm), the PEG moiety (3.46–3.64 ppm) and the Chol moiety (0.86–0.82, 0.62–0.69 ppm) (Additional file 1: Fig. S2).

### Characterization of different Dex preparations and incorporation of FA-PEG-Chol into Exo/Dex

**Fig. 2:**
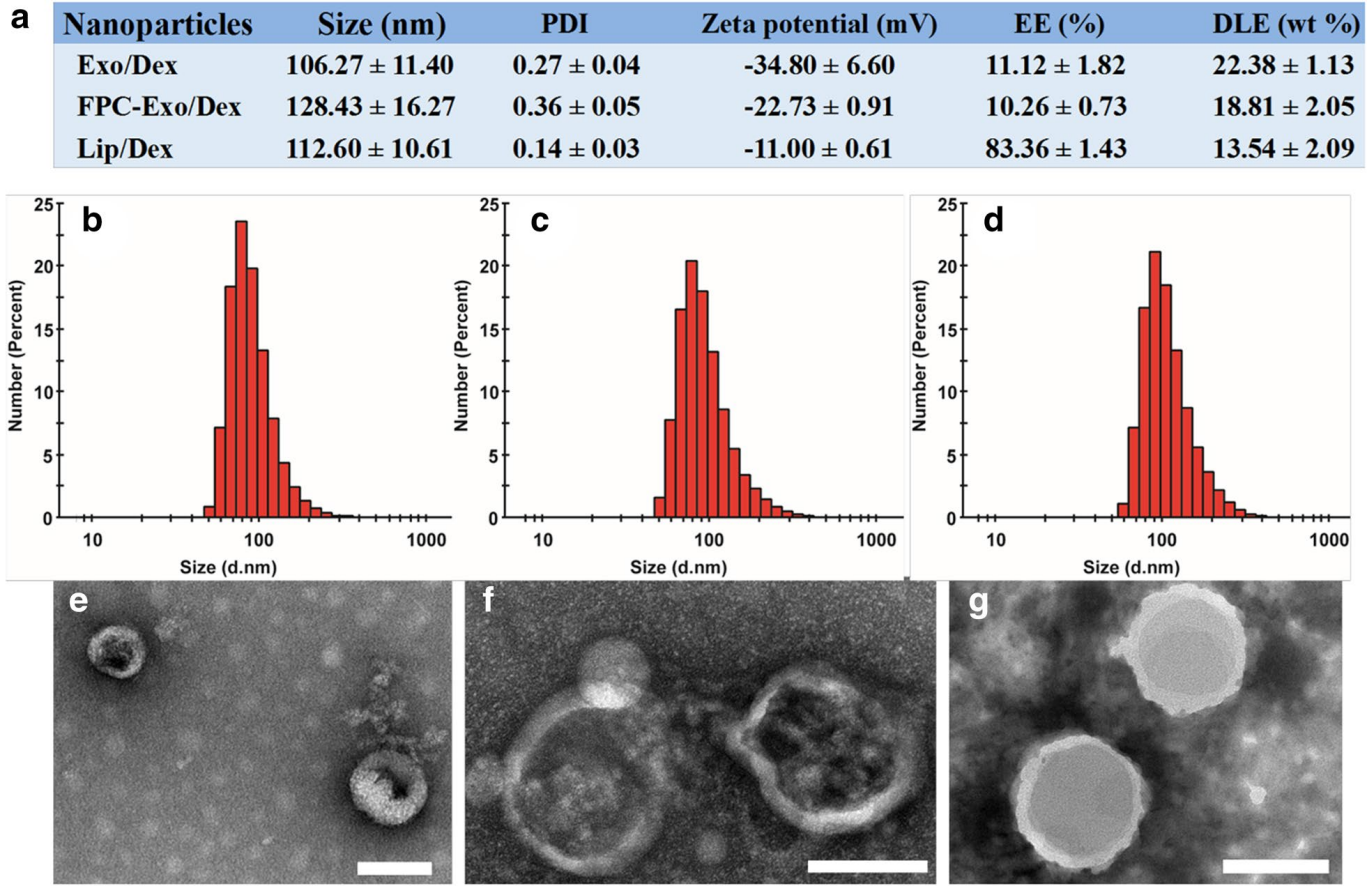
Characterization of Exo/Dex, FPC-Exo/Dex and Lip/Dex. **a** Size, PDI, zeta potential, EE and DLE of Dex preparations. Size distribution of Exo/Dex **b**, FPC-Exo/Dex **c** and Lip/Dex **d**. Transmission electron micrographs of Exo/Dex **e**, FPC-Exo/Dex ** f** and Lip/Dex **g**

Average diameter was smallest for Exo/Dex (106.27 ± 11.40 nm), intermediate for Lip/Dex (112.60 ± 10.61 nm) and largest for FPC-Exo/Dex (128.43 ± 16.27 nm) (Fig. 2a–d). Nevertheless, the three formulations did not differ significantly in size. Zeta potential was − 34.80 ± 6.60 mV for Exo/Dex, − 11.00 ± 0.61 mV for Lip/Dex and − 22.73 ± 0.91 mV for FPC-Exo/Dex (Fig. 2a). PDI was approximately 0.3 for all three formulations, indicating narrow size distribution. Transmission electron microscopy showed that Exo/Dex, FPC-Exo/Dex and Lip/Dex all exhibited typical sphere-like shapes (Fig. 2e–g). We used UV spectroscopy to determine that 21.37 ± 5.46 μg of FA was incorporated into 100 μg of exosomes.

Exo/Dex showed EE of 11.12 ± 1.82% and DLE of 22.38 ± 1.13%. The corresponding values for FPC-Exo/Dex were lower (10.26 ± 0.73% and 18.81 ± 2.05%), as were the values for Lip/Dex (83.36 ± 1.43% and 13.54 ± 2.09%) (Fig. 2a).

### In vitro cumulative drug release and toxicity of nanoparticles

**Fig. 3:**
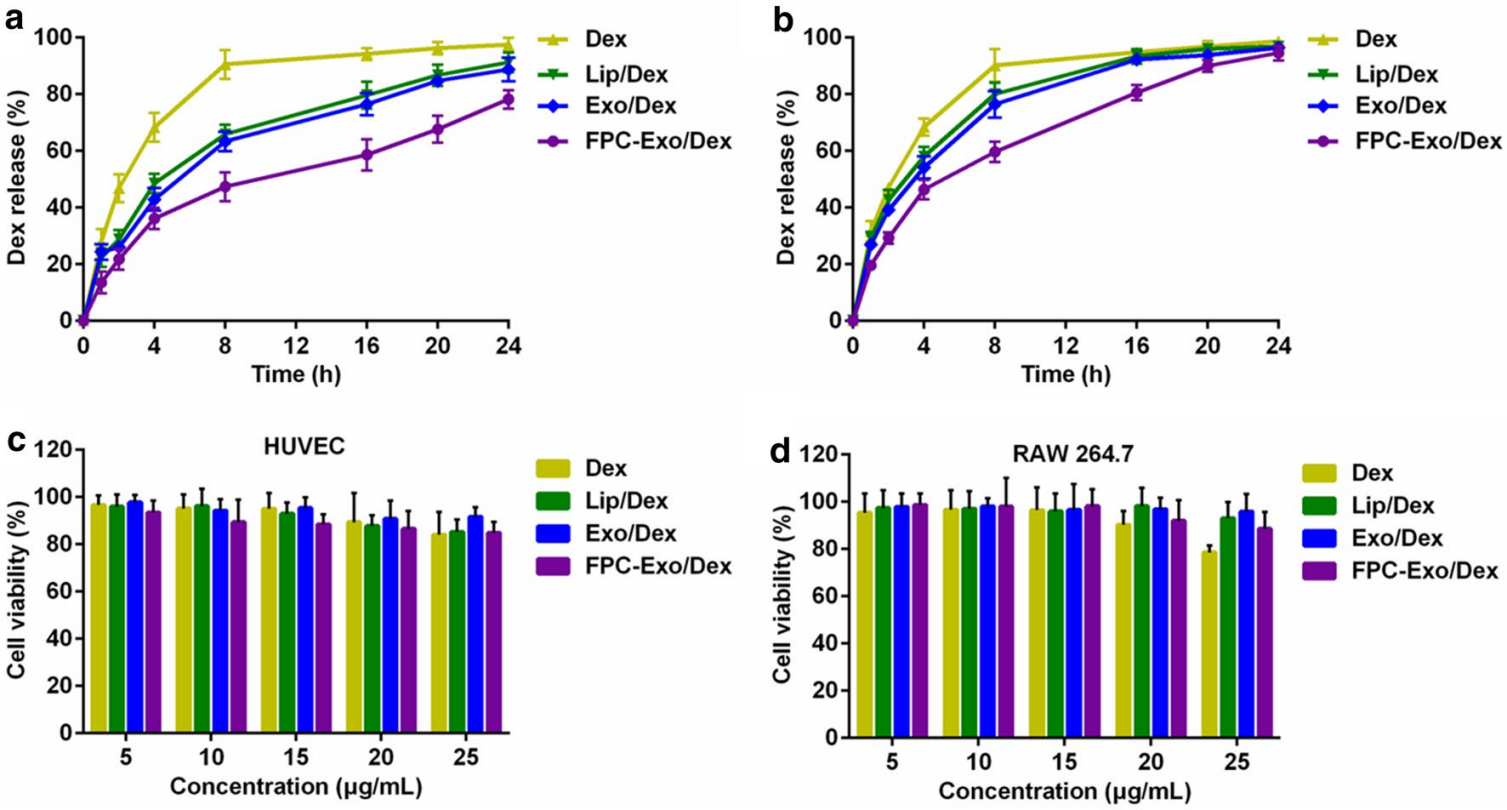
In vitro cumulative release and toxicity of Dex preparations. Cumulative release of Dex from Lip/Dex, Exo/Dex and FPC-Exo/Dex at PBS of pH 7.4 **a** or pH 6.0 **b**. Results were shown as mean ± SD (n = 3). The effect of Dex, Lip/Dex, Exo/Dex and FPC-Exo/Dex for HUVEC **c** and RAW264.7 cells viability **d**. Data represent mean ± SD (n = 5)

Dex was released from FPC-Exo/Dex in a slow, sustained fashion at pH 7.4 (Fig. 3a). During the first 8 h, only 47.26% of Dex was released from FPC-Exo/Dex, compared to 90.46% of Dex from the free drug solution. Drug release from Exo/Dex and Lip/Dex was intermediate between these two extremes. Under acidic conditions of pH 6.0, nearly 80% of encapsulated drug from FPC-Exo/Dex was released by 16 h (Fig. 3b). However, less than 60% released within 16 h at pH 7.4 (Fig. 3a), suggesting acidic pH triggered faster drug release from preparations.

Exposing HUVEC cultures for 24 h to free Dex or any of the Dex-loading formulations had no obvious effect on viability, which remained above 80% even at a concentration of 25 μg/mL (Fig. 3c). When we performed these toxicity experiments on RAW264.7 cultures, we found no significant decrease in viability with FPC-Exo/Dex, and a slight decrease with free Dex (Fig. 3d).

### Cellular uptake of nanoparticles by RAW264.7 cells

**Fig. 4:**
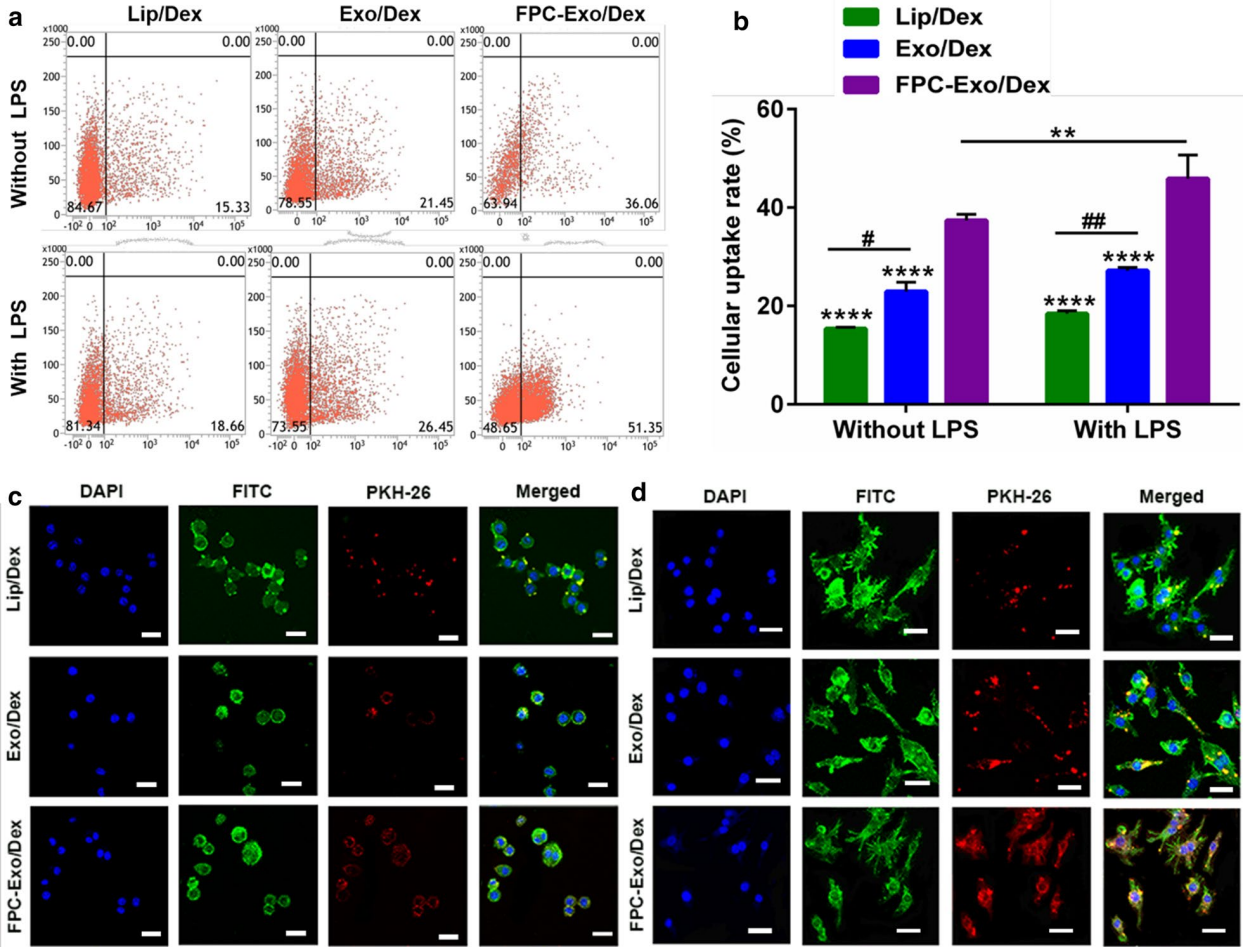
Uptake of Lip/Dex, Exo/Dex and FPC-Exo/Dex by RAW264.7 with or without LPS activation. **a** Representative images of Flow cytometry analysis showing uptake of Dex preparations labeled by PKH 67 in RAW264.7 cells with or without LPS activation. **b** Uptake rate of different Dex preparations by flow cytometry (n = 3, **p < 0.01, ****p < 0.0001 vs cells treated with FPC-Exo/Dex, ^#^*p* < 0.05, ^##^*p* < 0.01 are cells treated with Lip/Dex compared to Exo/Dex). **c** Confocal microscopy showing uptake of different Dex preparations labeled by PKH 67 in RAW264.7 cells without LPS activation. Dex preparations appear in red; nucleus, blue; and cytoplasm, green. Scale bar, 20 μm. **d** Confocal microscopy showing uptake of different Dex preparations labeled by PKH 67 in RAW264.7 cells without LPS activation. Dex preparations appear in red; nucleus, blue; and cytoplasm, green. Scale bar, 20 μm

RAW264.7 cells internalized larger amounts of Dex formulations after activation with LPS, especially the largest of FPC-Exo/Dex (Fig. 4a, b). Regardless of activation status, cells took up more FPC-Exo/Dex than the other Dex formulations. Moreover, the endocytosis of Exo/Dex was more than Lip/Dex. These flow cytometry results were supported by confocal imaging (Fig. 4c, d). Confocal images instruct that FPC-Exo/Dex entered the cytoplasm the most, followed by Exo/Dex, especially in LPS-activated RAW264.7 cells.

### Effect to inflammatory cytokines secretion

**Fig. 5:**
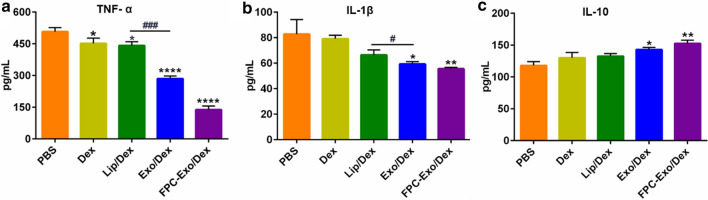
Expression of inflammatory cytokines in cell-free supernatants treated with different preparations. Level of **a** TNF-α, **b** IL-1β, and **c** IL-10 (n = 3, *p < 0.05, **p < 0.01, ***p < 0.001 vs cells treated with PBS, ^#^*p* < 0.05, ^###^*p* < 0.001 are cells treated with Lip/Dex compared to Exo/Dex)

TNF-α levels in culture medium were much lower after LPS-activated RAW264.7 cells were treated with FPC-Exo/Dex and Exo/Dex than with free Dex or Lip/Dex (Fig. 5a). Similarly, the formulations inhibited secretion of IL-1β, with FPC-Exo/Dex showing a stronger effect, then followed by Exo/Dex (Fig. 5b), suggesting that the exosome-based drug delivery system exerts better anti-inflammatory effects. FPC-Exo/Dex significantly up-regulated the anti-inflammatory cytokine IL-10 (*p* < 0.01), Exo/Dex also did (*p* < 0.05) (Fig. 5c).

### Biodistribution of nanoparticles in CIA mice

**Fig. 6:**
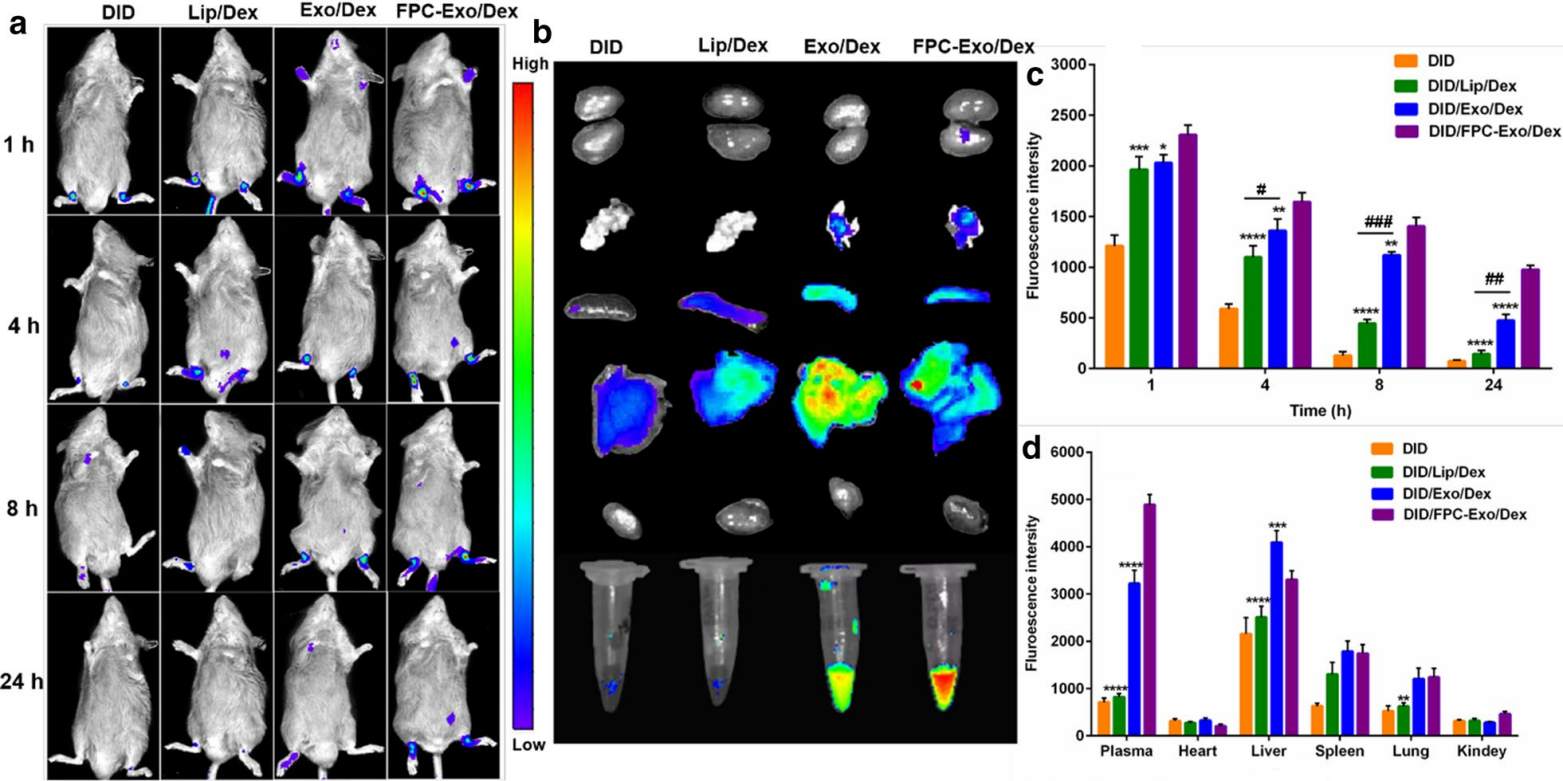
The real-time fluorescence imaging of CIA mice. **a** Real-time fluorescence imaging of CIA mice after intravenous injection with free DID, DID/Lip/Dex, DID/Exo/Dex and DID/FPC-Exo/Dex, respectively (n = 3). **b** Ex vivo imaging of the plasma and organs at 24 h after intravenous injection. **c** Semi-quantitation of fluorescence intensity in joints. **d** Semi-quantitation of fluorescence intensity in joints in plasma and organs (n = 3, *p < 0.05, **p < 0.01, ***p < 0.001, ****p < 0.0001 vs mice injectedted with DID/FPC-Exo/Dex, ^#^*p* < 0.05, ^##^*p* < 0.01, ^###^*p* < 0.001 are mice injected with DID/Lip/Dex compared to DID/Exo/Dex)

Real-time fluorescence imaging revealed that all three DID-labeled preparations accumulated in the joints of CIA mice at 1 h (Fig. 6a), presumably due to the ELVIS effect. DID/FPC-Exo/Dex showed the greatest accumulation into joints at every time point. Even at 24 h, the DID/FPC-Exo/Dex group showed intensive fluorescence, whereas the DID/Lip/Dex group showed slight signal by 8 h (Fig. 6a). Semi-quantitation of fluorescence intensity in ankle joints further indicate that DID/FPC-Exo/Dex group displayed more significant fluorescence than other groups (Fig. 6c). In addition, DID/Exo/Dex group displayed significantly fluorescence at 4, 8 and 24 h compare to DID/Lip/Dex group (Fig. 6c). These results suggest that FPC-Exo/Dex may target inflammatory lesions better and persist there longer than Exo/Dex and Lip/Dex, and Exo/Dex showed better targeting performance than Lip/Dex. Ex vivo imaging indicated that DID/FPC-Exo/Dex accumulated more in plasma than other groups after 24 h, suggesting its long systemic circulation (Fig. 6b, d). Furthermore, obvious fluorescence was detected in livers of all groups, however, DID/FPC-Exo/Dex group had lower distribution than Exo/Dex (Fig. 6b, d), suggesting modifying with FPC could decrease non-specific distribution. Fluorescence in spleens and lungs was faint, and almost no fluorescence was detected in kidney (Fig. 6b, d).

### Therapeutic efficacy in CIA mice based on body weight, AI scores, paw thickness and foot volume

**Fig. 7:**
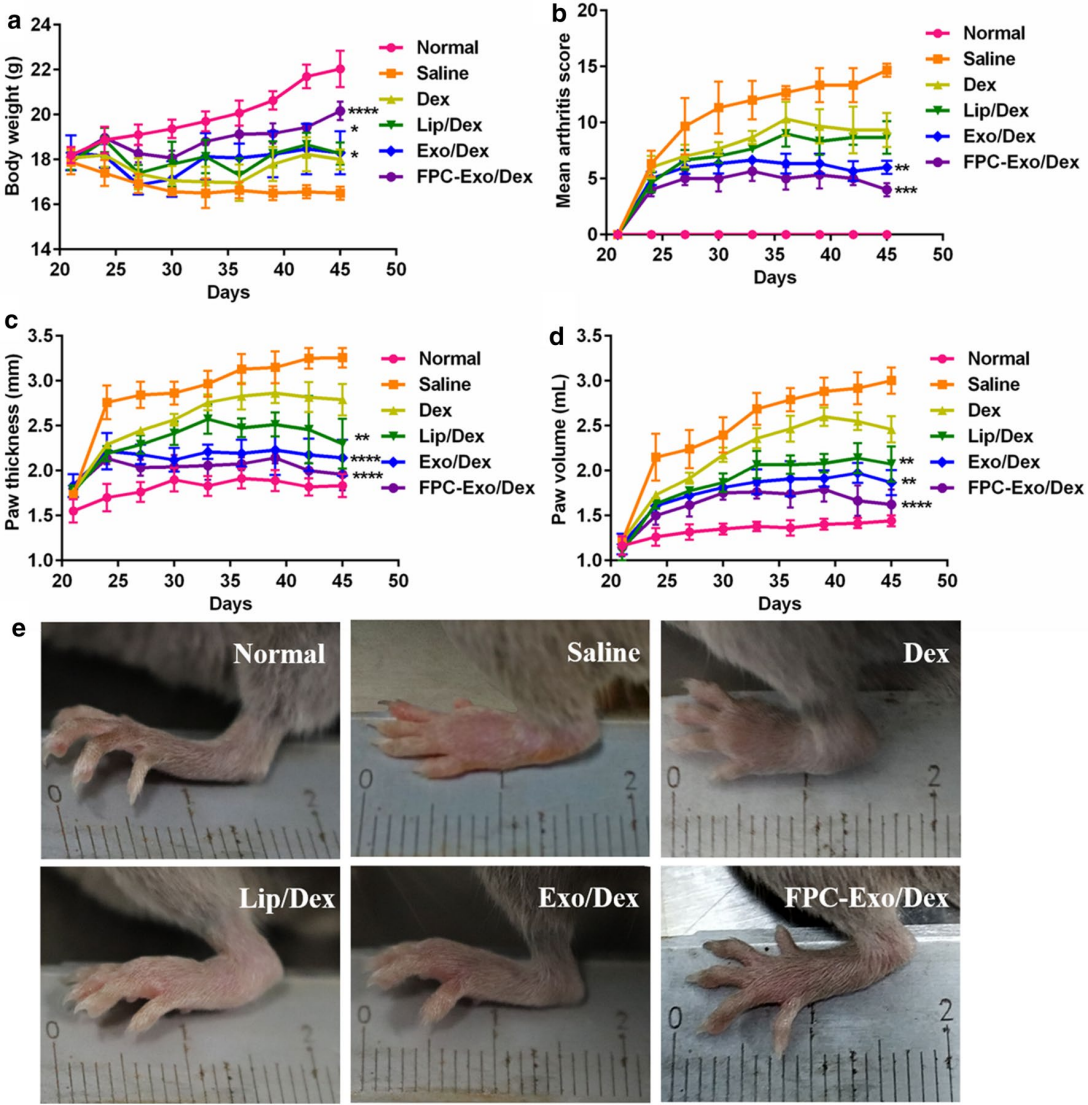
Therapeutic indicators monitoring during treatment. Measurement of body weight **a**, articular score **b**, paw thickness**c**, paw volume **d** and photographs of representative hind limbs from animals treated with various Dex formulations in CIA mice **e**. (n = 3, *p < 0.05, **p < 0.01, ***p < 0.001, ****p < 0.0001 vs mice injected with Saline)

Body weight serves as an indirect indicator of therapeutic efficacy in RA since disease progression is associated with weight loss due to less feeding and greater apathy [33]. Body weight in mice injected with FPC-Exo/Dex increased continuously from day 21 (2nd booster injection) until day 45, and it had no significant difference with Normal group (Fig. 7a). All treatment groups showed an initial increase in scores, reflecting disease progression, followed by a decrease in scores, reflecting therapeutic effects (Fig. 7b). FPC-Exo/Dex was associated with the lowest AI scores (Fig. 7b). This formulation also led to the least swelling of the hind limbs, based on paw thickness and foot volume, followed by Exo/Dex (Fig. 7c, d).

Imaging of the left hind limbs in animals treated with saline showed severe, extensive joint swelling and joint deformation, which free Dex reduced slightly but not markedly (Fig. 7e). FPC-Exo/Dex reduced this swelling nearly completely, such that the difference from saline-treated animals was not significant. Exo/Dex also significantly ameliorated swelling, but not as much as FPC-Exo/Dex.

### Microstructure of articular bone based on Micro-CT

**Fig. 8:**
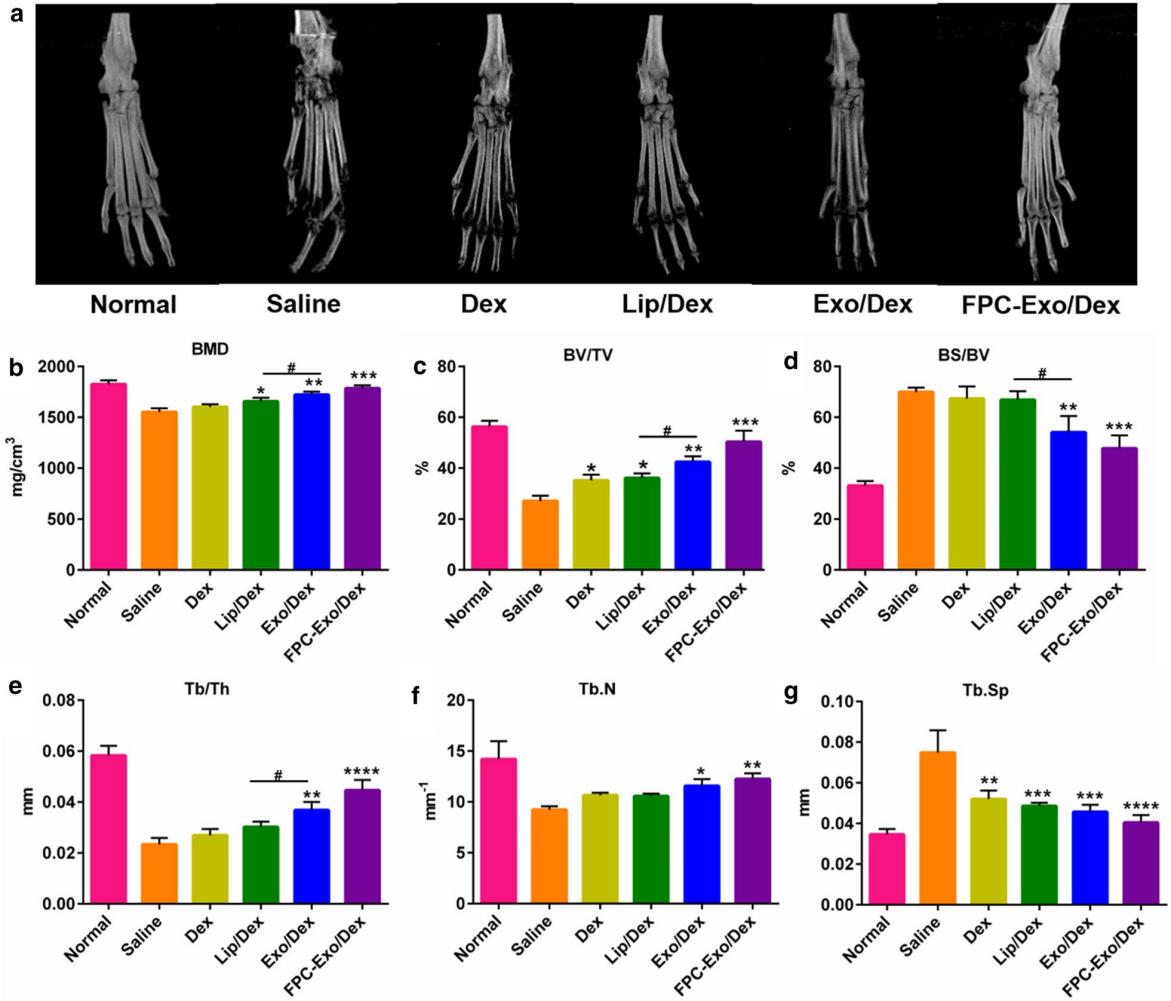
Micro-CT analyses of the hind limbs of the mice from different treatment groups. **a** Representative 3D reconstructed images from each treatment group. **b**–**g** Bone morphometric parameters of ROI within calcaneus bone (n = 3, *p < 0.05, **p < 0.01, ***p < 0.001, ****p < 0.0001 vs mice treated with Saline, ^#^*p* < 0.05 is mice treated with Lip/Dex compared to Exo/Dex)

Saline-treated animals showed the most severe damage, with extensive erosion of the bone in ankle and toe joints (Fig. 8a). FPC-Exo/Dex was associated with significantly lower ankle bone erosion, which was no longer obvious after four times administrations. Quantitative analysis of the ROI in calcaneus showed that FPC-Exo/Dex treatment preserved the bone quality as evident in the morphometric parameters, such as bone mineral density (BMD), percent bone volume (BV/TV), bone surface density (BS/BV), trabecular thickness (Tb.Th), trabecular number (Tb.N) and trabecular spacing (Tb.Sp), with their values similar to those observed for healthy controls; significantly better than those observed for other Dex formulations treated groups (Fig. 8b–g). Meanwhile, the values of BMD, BV/TV, BS/BV and Tb.Th in Exo/Dex-treated group are significantly lower than these of Lip/Dex-treated groups, and Exo/Dex-treated group displayed a slightly lower articular bone erosion.

### Histological analysis of joint tissue

**Fig. 9:**
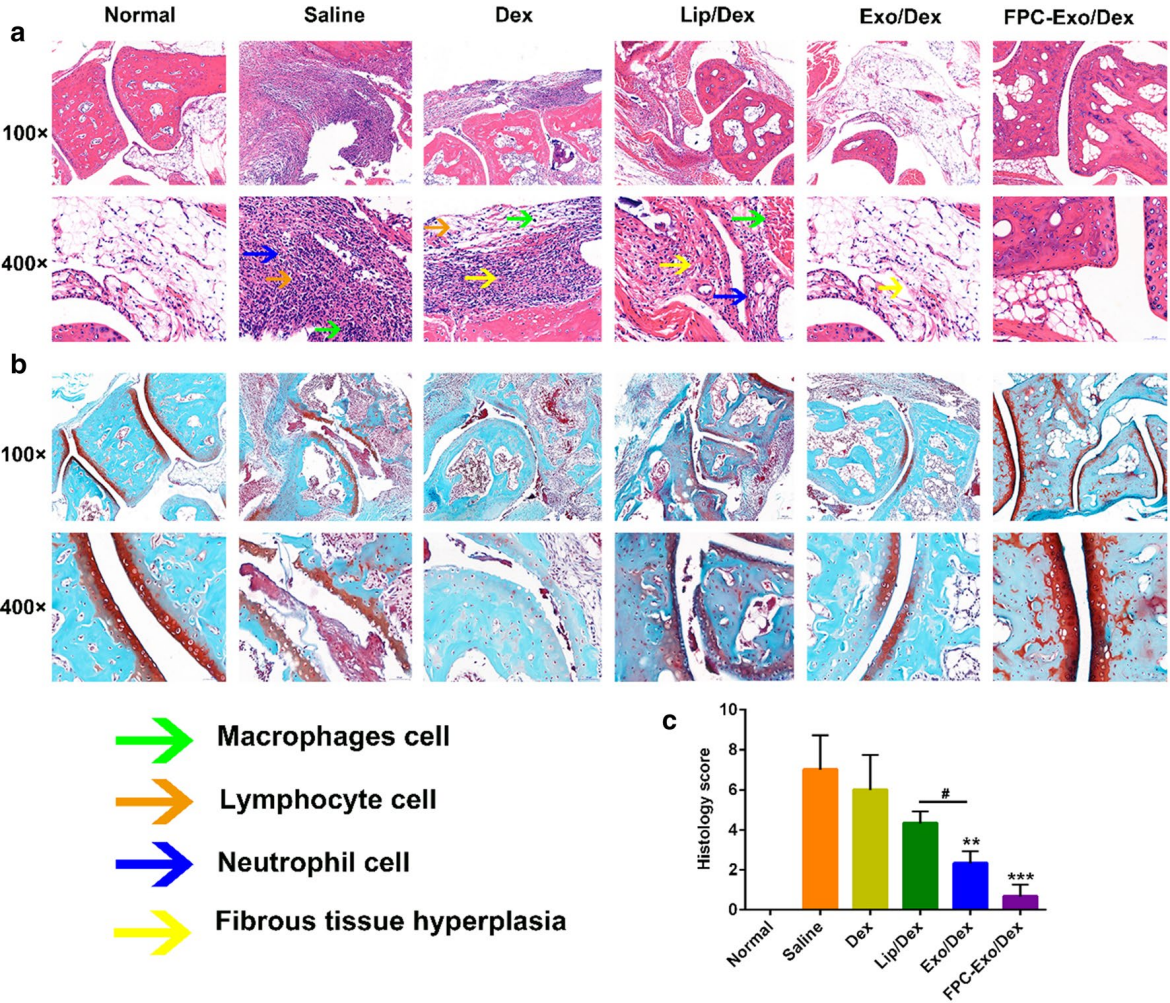
Histopathology analysis of the ankle joints. **a** Representative histopathology pictures of H&E staining. **b** Representative histopathology pictures of SO staining. **c** Histopathology scores of H&E staining (n = 3, **p < 0.01, ***p < 0.001 vs mice treated with Saline, ^#^*p* < 0.05 is mice treated with Lip/Dex compared to with Exo/Dex)

Compared to healthy control animals, H&E staining of histology slices from CIA mice treated with saline or free Dex revealed marked inflammatory cell infiltration, synovial tissue expansion and fibrous tissue hyperplasia (Fig. 9a). Average histopathology scores were 7.0 in the Saline group and 6.0 in the Dex-treated group. Animals treated with Lip/Dex showed a score of 4.3, indicating moderate damage. The corresponding scores in the Exo/Dex and FPC-Exo/Dex groups were 2.3 and 0.7, indicating markedly reduced joint damage of FPC-Exo/Dex (Fig. 9c).

Based on SO staining, articular cartilage was nearly destroyed in animals treated with saline or free Dex (Fig. 9b), but the destruction was significantly less severe in animals treated with FPC-Exo/Dex. In addition, joints from animals treated with Lip/Dex showed weaker red staining on articular surfaces than Exo/Dex-treated animals, suggesting severer degradation and destruction of articular cartilage tissue. Qualitative analysis suggested that FPC-Exo/Dex protected the cartilage to the greatest extent.

### Inflammatory Cytokines in serum of CIA mice after treatment

**Fig. 10:**
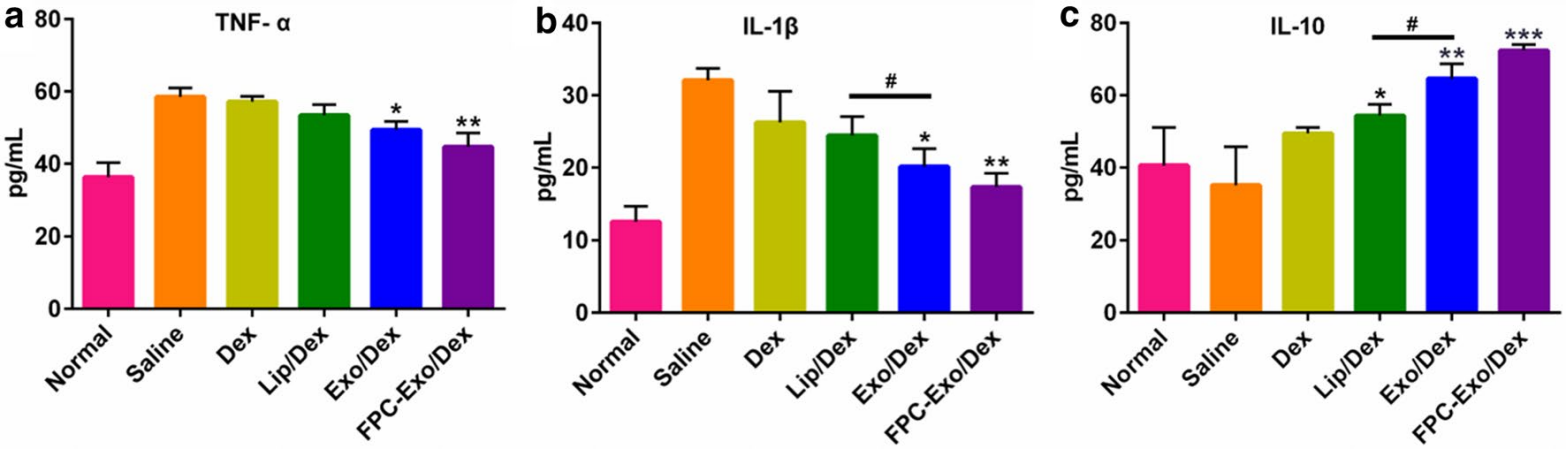
Levels of the inflammatory cytokines in serum. Level of TNF-α **a**, IL-1β **b**, and IL-10 **c**, results are shown as mean ± SD (n = 3, *p < 0.05, **p < 0.01, ***p < 0.001 vs mice treated with Saline, ^#^*p* < 0.05 is mice treated with Lip/Dex compared to Exo/Dex)

Following CIA induction, levels of both TNF-α and IL-1β in the serum increased, and these increases were marginally but not significantly smaller with free Dex or Lip/Dex, while significantly smaller with FPC-Exo/Dex (*p* < 0.01) and Exo/Dex (*p* < 0.05) were observed (Fig. 10a, b). In addition, the level of IL-1β in Exo/Dex-treated group is lower than it of Lip/Dex-treated group. Meanwhile, FPC-Exo/Dex treatment increased the level of IL-10 the most in all groups (*p* < 0.001), then Exo/Dex (*p* < 0.01) (Fig. 10c).

### Hepatotoxicity analysis of nanoparticles in CIA mice after treatment

**Fig. 11:**
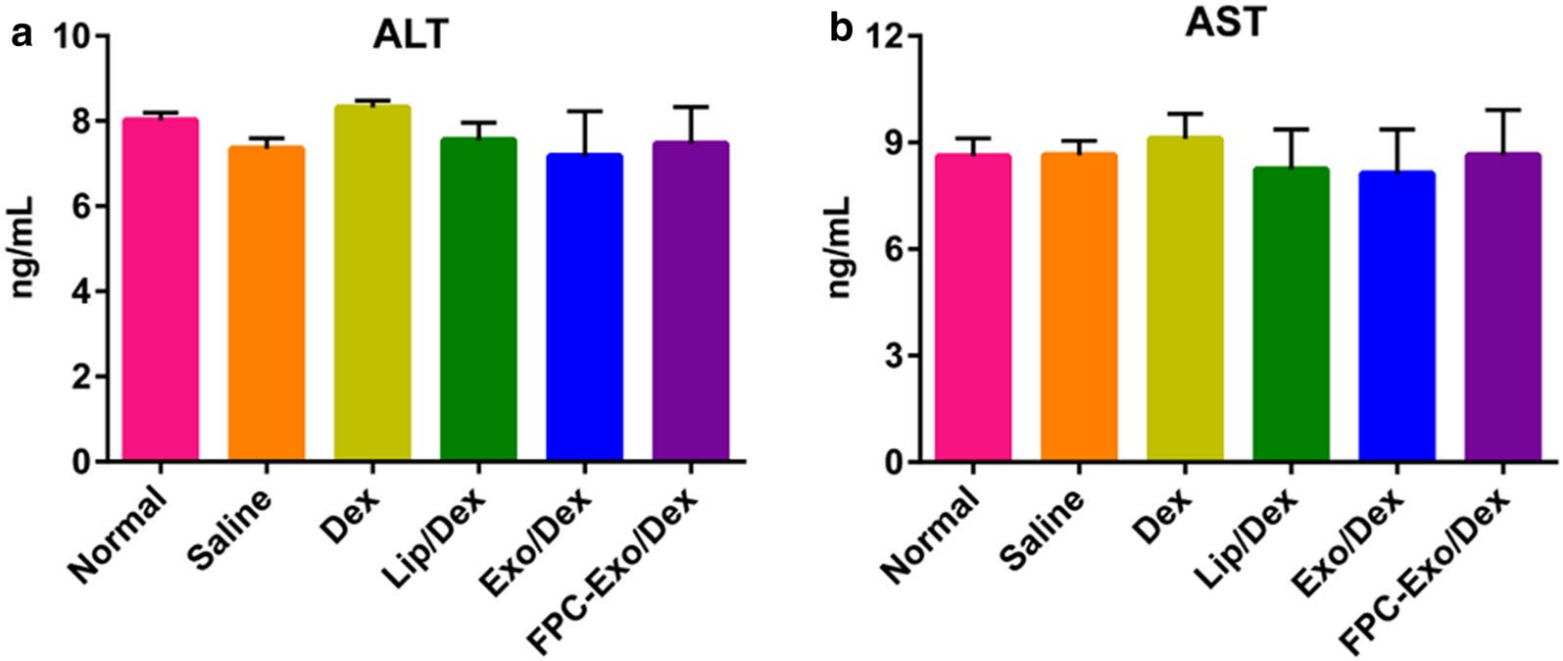
Levels of AST and ALT in serum (n = 3). All the treatment groups were compared to Normal group

Serum levels of AST and ALT in any of the Dex-treated groups were not significantly different from those in the healthy, untreated controls (Fig. 11), indicating that this therapeutic strategy may not cause obvious damage to the liver. FPC-Exo/Dex was associated with marginally but not significantly levels of both indices than free Dex (Fig. 11). These results suggest desirable biocompatibility of FPC-Exo/Dex in vivo.

## Discussion

In this study, we derived the exosomes from RAW264.7 macrophages because inflammatory lesions in RA contain abundant activated macrophages, and target cells appear to internalize exosomes better when these exosomes have been derived from the same cell type [26, 34–36]. Furthermore, it was reported that exosomes derived from macrophages have achieved excellent therapeutic efficacy in the treatment of other inflammations [20, 22]. Therefore, the choice of macrophages as the donor cell of exosomes is cogitative. We extract exosomes using ultracentrifugation because this classical approach may extract exosomes more efficiently and provide better dispersity than other extraction methods [37, 38]. We substantially shortened the ultracentrifugation time by adding an initial ultrafiltration to reduce the volume of the medium before the second ultracentrifugation. Dynamic light scattering showed the resulting exosomes to be uniformly disperse in the narrow range of 80–110 nm, and a protein assay estimated a yield of 2.20 ± 0.44 μg of exosomes from 1 mL of culture medium. These results indicate that our combination of ultrafiltration and ultracentrifugation is suitable for the extraction of exosomes from RAW264.7 cells with adequate yields for subsequent experiments.

Electroporation is widely used to load chemical drug, short interfering RNA and DNA into exosomes [39, 40]. We included 80 mM trehalose in the mixture of Dex and exosomes during electroporation to prevent aggregation of the exosomes [41], which may help explain why we obtained a uniform dispersion of size with narrow PDI.

We modified the exosomes with FA-PEG-Chol compound (FPC) to optimize their properties for better treating of RA, for folic acid can help the particles accumulate and persist in inflamed tissues, where abundant activated macrophages express FR [3]. Moreover, PEG can enhance stability and long circulation time of drug delivery system [42], and both the PEG and Chol help solubilize the FA and ensure its stable incorporation into the membrane of the exosomes. In vitro cumulative drug release study indicated that Dex was released more sustainably from FPC-Exo/Dex than from Exo/Dex or free Dex. Biodistribution examination further showed that FPC-Exo/Dex was retained more and longer in joint tissues than Exo/Dex in CIA mice. These results confirm that our “FPC” modification of Dex-loaded exosomes ensured their active targeting to inflammatory sites in RA, but also due to the fact that PEG can prolong circulation time. Consistent with this, cellular uptake studies also indicted that FPC-Exo/Dex was taken up by LPS-activated macrophages to a greater extent than Exo/Dex or Lip/Dex.

Dex and other glucocorticoids are used to treat RA because they down-regulate secretion of pro-inflammatory cytokines such as IL-1β, TNF-α and IL-6 by activated macrophages at inflammation lesions, while up-regulating secretion of the anti-inflammatory cytokine IL-10 [10, 11, 19]. We found that FPC-Exo/Dex triggered these therapeutic effects in CIA mice to a significantly greater extent than other Dex formulations. These effects correlated with significantly lower histopathology scores based on H&E staining analysis, significantly less cartilage and articular bone destruction. based on SO staining and micro-computed tomography analysis. The less severe bone destruction may be an indirect result of the ability of FPC-Exo/Dex to reduce secretion of IL-1β and TNF-α, since these factors may promote the formation of osteoclasts [43–45]; and to promote secretion of IL-10, which can inhibit osteoclast formation. The superior therapeutic effects of FPC-Exo/Dex over the other Dex formulations may relate to its better targeting and internalization.

FPC-Exo/Dex showed good biocompatibility not only in culture studies with HUVEC and RAW264.7 cells but also in mice. Exosomes tend to accumulate in the liver after intravenous administration [46, 47], and biodistribution assay showed substantial accumulation of Exo/Dex and FPC-Exo/Dex in liver. However, there was no significant difference between Exo/Dex or FPC-Exo/Dex group and normal group for the liver function indices AST and ALT, suggesting that encapsulating Dex into exosomes did not cause hepatotoxicity.

Results of comparative research between Exo/Dex and Lip/Dex suggest that exosome-based on drug delivery system had better internalization, weaker blood clearance and long-term circulating capability. Consistently, Exo/Dex displayed better therapeutic efficacy than Lip/Dex, reflecting the advantages of exosome as an endogenous nanocarrier over liposome.

Although exosome is an idea nanocarrier to delivery GCs for treatment to RA, the encapsulation efficiency of exosome-based drug system is mostly less than 30% [22, 48], which is much lower than the encapsulation efficiency in physical nanoparticles (almost over 90%) [8, 49]. It has been reported that encapsulating drug-loading physical nanoparticles into exosomes could enhance the encapsulation efficiency of drug delivery system and take endogenous advantage of exosomes [50, 51], which also provides new idea for our future research.

## Conclusion

We prepared relatively simple, biocompatible FPC-Exo/Dex nanoparticles as a drug delivery platform. These particles show the advantages of other formulations as well as greater stability and longer persistence because of their PEG-Chol-FA modification. These modified exosomes were endocytosed better in vitro than Exo/Dex, or free Dex, and they targeted inflamed joints and protected bone and cartilage better in mice with collagen-induced arthritis. They down-regulated the levels of pro-inflammatory cytokines and up-regulated anti-inflammatory cytokine, strongly inhibiting macrophage-driven inflammation in CIA mice. The FPC-Exo/Dex system constructed in our study may be a useful drug delivery system for GCs to treat RA.

## Supplementary information

**Additional file 1. Fig. S1.** 1H NMR spectra of FA-NHS.** Fig. S2.** 1H NMR spectra of FA-PEG-Chol.** Fig. S3.** The formation of FA-NHS ester.** Fig. S4.** The reaction scheme of FA-NHS with Chol-PEG-NH2.

## Data Availability

All data generated or analyzed during this study are included in this article.

## Abbreviations

GCs: Glucocorticoids
RA: Rheumatoid arthritis
Exo: Exosome
FA: Folic acid
Dex: Dexamethasone sodium phosphate
PEG: Polyethylene glycol
Chol: Cholesterol
ELISA: Enzyme-linked immunosorbent assay
CIA: Collagen-induced arthritis
EE: Encapsulation efficiency
DLE: Drug loading efficiency
ELVIS: Extravasation through leaky vasculature and subsequent inflammatory cell-mediated sequestration
Lip: Liposome
PDI: Polydispersity index
TEM: Transmission electron microscopy
HUVEC: Human umbilical vein endothelial cells
MTT: 3-(4,5dimethylthiozol-2-yl)-2,5-diphenyl-tetrazolium bromide
LPS: Lipopolysaccharide
Micro-CT: Micro-computed tomography
DID: 1,1′-dioctadecyl-3,3,3′,3′-etramethylindodicarbocyanine perchlorate
AI: Articular index
H&E: Hematoxylin–eosin staining
SO: Safranin O staining
AST: Aspartate aminotransferase
ALT: Alanine transaminase
