# Supplementary material for: Exosome-based biomimetic nanoparticles targeted to inflamed joints for enhanced treatment of rheumatoid arthritis
Source: J Nanobiotechnology. 2020 Aug 20;18:115. doi: 10.1186/s12951-020-00675-6 (PMC7441703; doi:10.1186/s12951-020-00675-6)
Supplement: Supplementary file 1 — Additional file 1. Fig. S1. 1H NMR spectra of FA-NHS. Fig. S2. 1H NMR spectra of FA-PEG-Chol. Fig. S3. The formation of FA-NHS ester. Fig. S4. The reaction scheme of FA-NHS with Chol-PEG-NH2. [file 12951_2020_675_MOESM1_ESM.docx]

Additional file

**Exosome-based biomimetic nanoparticles targeted to inflamed joints for enhanced treatment of rheumatoid arthritis**

Yan Feili ^#1^, Zhong Zhirong ^#1^, Wang Yao ^#1^, Feng Yue ^2,3^, Mei Zhiqiang ^4^, Li Hui ^1^, Chen Xiang ^1^, Cai Liang *^2,3^, Li Chunhong *^1,5^

^1^Department of Pharmaceutical Sciences, School of Pharmacy, Southwest Medical University, Luzhou 646000, Sichuan, China.

^2^Department of N[uclear](javascript:void(0);) [medicine](javascript:void(0);), The Affiliated Hospital of Southwest Medical University, Luzhou, Sichuan, 646000, China.

^3^Nuclear Medicine and Molecular Imaging Key Laboratory of Sichuan Province, Luzhou, Sichuan, 646000, China.

^4^The Research Center for Preclinical Medicine, Southwest Medical University, Luzhou, Sichuan 646000, China.

^5^Engineering Research Center in Biomaterials, Sichuan University, Chengdu, Sichuan, 610064, P. R. China.

Corresponding author: Cai Liang

E-mail: cllc131420@sina.com

Tel: +86 18982702720

Fax numbers: +86 08306302050

Mail address: Department of N[uclear](javascript:void(0);) [medicine](javascript:void(0);), The Affiliated Hospital of Southwest Medical University, 3-319 Zhongshan Road, Luzhou, Sichuan 646000, People’s Republic of China.

Corresponding author: Li Chunhong

E-mail: [lispringhong@126.com](mailto:lispringhong@126.com)

Tel: +86 13679696586

Fax numbers: +86 08306302050

Mail address: Department of Pharmaceutical Sciences, School of Pharmacy, Southwest Medical University, 3-319 Zhongshan Road, Luzhou, Sichuan 646000, People’s Republic of China.

Yan Feili, Zhong Zhirong and Wang Yao contributed equally to this work.

**SUPPLEMENTARY SCHEMES AND FIGURES**

**Fig. S1**


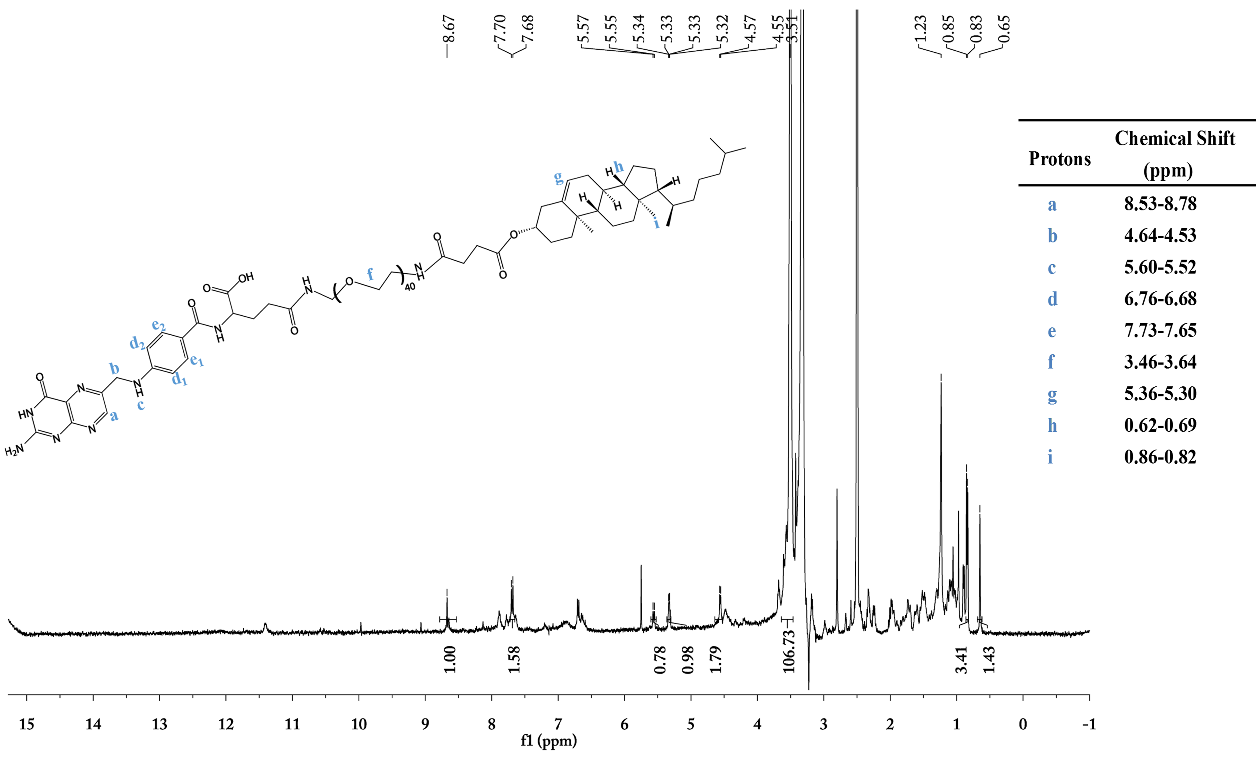


**Fig. S2**


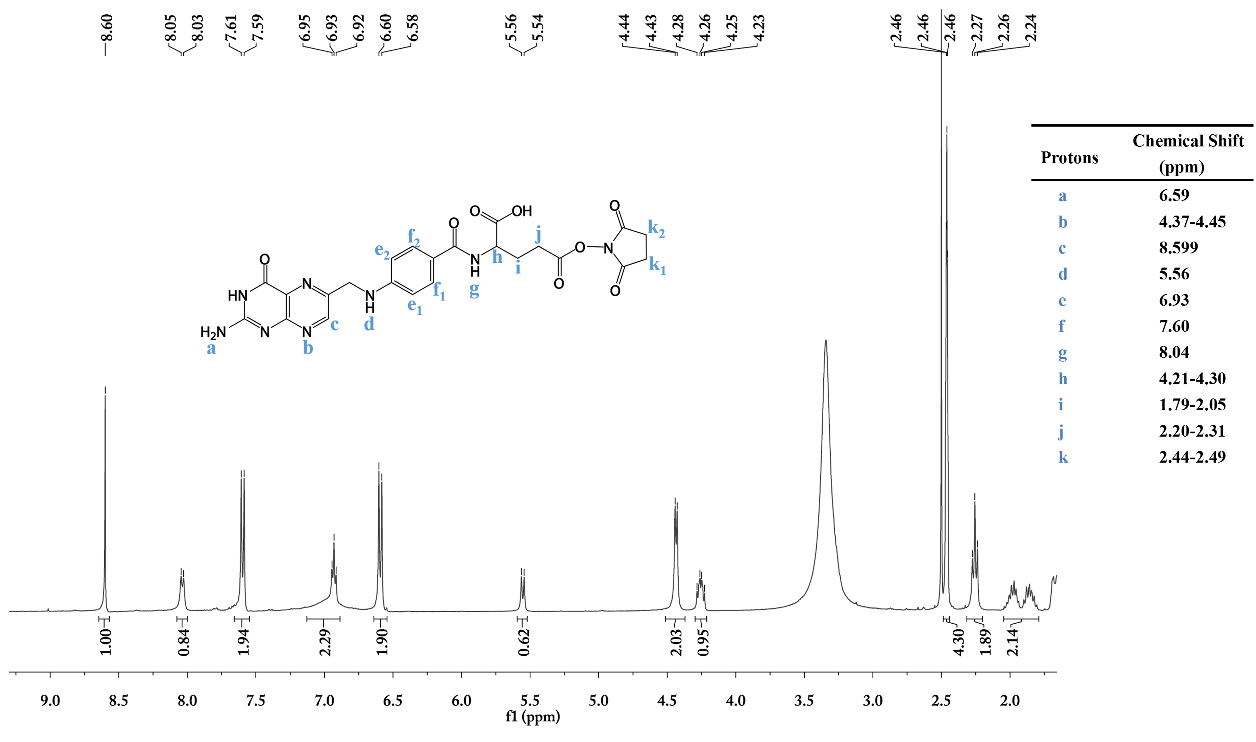


**SUPPLEMENTARY METHOD**

**Materials**

Folic acid (FA) was supplied by the Solarbio science & Technology Co., Ltd (Beijing, China). N-hydroxysuccinimide (NHS) and dicyclohexylcarbodiimide (DCC) were purchased from Chengdu Runze Local Chemical Co., Ltd (Chengdu, China). Chol-PEG-NH2 (MWCO 2000 Da) was obtained from Melo Technology (Shenzheng China). Others chemical reagents and solvents were of analytic grade.

**Synthesis of FA-NHS Ester**

N-hydroxysucci-mide (NHS) and dicyclohexylcarbodiimide (DCC) were used to activate the carboxylate group of FA [1]. Briefly, 1 g of FA was dissolved in 50 mL dimethyl sulfoxide (DMSO). Then, 1.2 ratio molar excess of NHS and DCC were added to the solution, which was stirred for 24 h at room temperature under shielding from light. Next, the by-product was removed by filtration and supernatant was freeze-dried. Finally, ^1^H-NMR of FA-NHS ester was analyzed by Nuclear Magnetic Resonance Spectra (400 MHz, DMSO-d6).

**Figure S3**


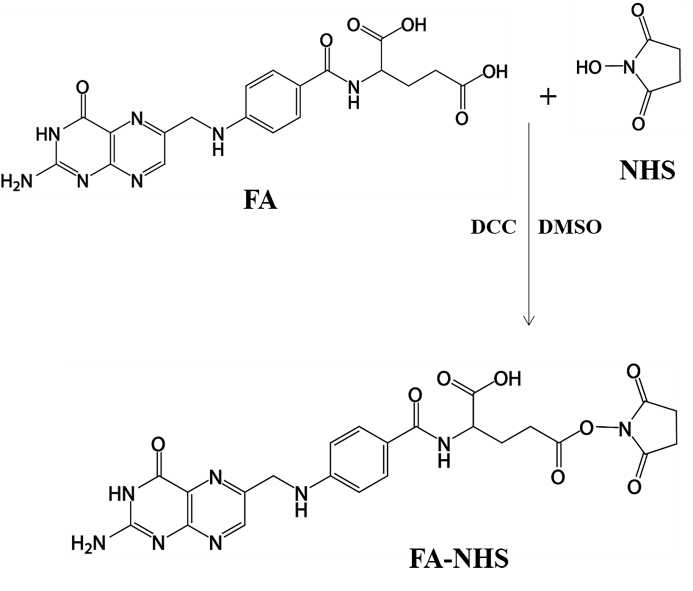


**Preparation of FA-PEG-Chol Conjugate**

Furthermore, FA-NHS ester was reacted with PEG-Chol-NH_2_ to form FA-PEG-Chol as following method. FA-NHS, 161.5 mg (0.3 mm), mixed with 503 mg (0.2 mm）of Chol-PEG-NH_2_ was dissolved in 10 mL DMSO and reacted under nitrogen atmosphere at room temperature for 5 h [1,2]. Then 20 mL of acetone was added to dilute the resultant solution, which was further centrifuged at 4000 rpm for 10 min. Next, the supernatant was dialyzed against water for three times (MWCO 2000Da) and then it was freeze-dried [3]. Finally, the structure of FA-PEG-Chol was determined using ^1^H-NMR spectroscopy (400 MHz, DMSO-d6).

**Figure S4**


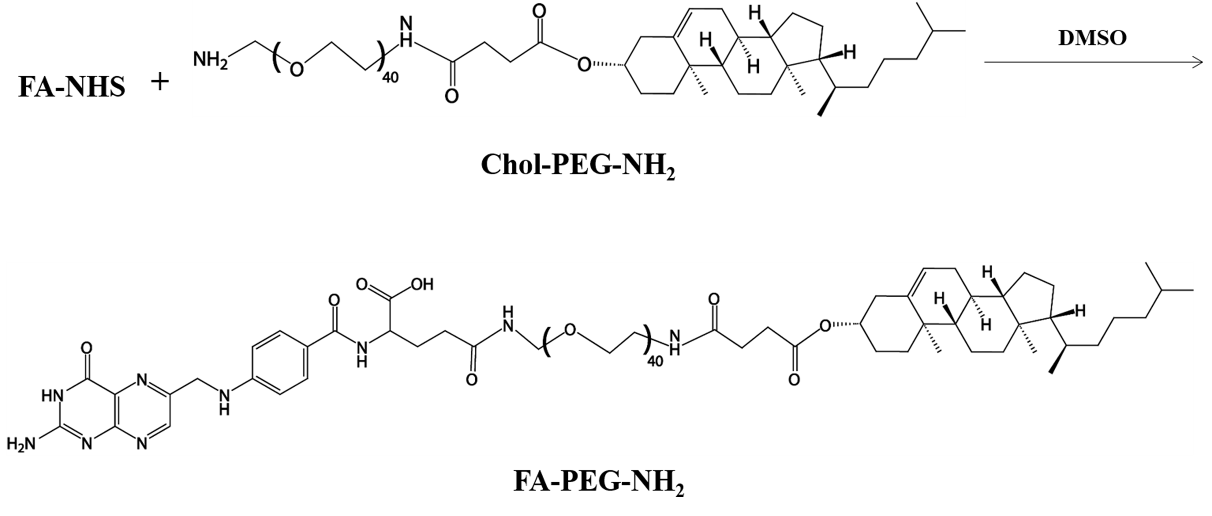


**REFERENCE FOR SUPPORT MATERIAS**

1. Güliz Ak, Sanlıer SH. Synthesis of folate receptor-targeted and doxorubicin-coupled chemotherapeutic nanoconjugate and research into its medical applications. Prep Biochem Biotechnol. 2013; 42: 551-563.
2. Puchkov PA, Shmendel EV, Luneva AS, Morozova NG, Zenkova MA, Maslov MA. Design, synthesis and transfection efficiency of a novel redox-sensitive polycationic amphiphile. Bioorg Med Chem Lett. 2016; 26: 5911-5915.
3. Malhi SS, Budhiraja A, Arora S, Chaudhari KR, Nepali K, Kumar R. Intracellular delivery of redox cycler-doxorubicin to the mitochondria of cancer cell by folate receptor targeted mitocancerotropic liposomes. Int J Pharm. 2012; 432: 63-74.
